# Impact of Adopting the Race/Ethnicity-Free 2021 CKD-EPI Equation on Cardiovascular Risk Stratification in a Multiethnic European Population: Results from the HELIUS Study

**DOI:** 10.5334/gh.1579

**Published:** 2026-08-21

**Authors:** Brechje J. M. V. Huisman, Taryn G. Vosters, Charles Agyemang, Bert-jan H. van den Born, Irene G. M. van Valkengoed, Liffert Vogt

**Affiliations:** 1 Department of Internal Medicine, Section Nephrology, Amsterdam UMC, University of Amsterdam, Amsterdam, The Netherlands; 2 Department of Internal Medicine, Slingeland Hospital, Doetinchem, The Netherlands; 3 Department of Public and Occupational Health, Amsterdam UMC, University of Amsterdam, Amsterdam Public Health (APH) Research Institute, Amsterdam, The Netherlands; 4 Department of Internal Medicine, Section Vascular Medicine, Amsterdam UMC, University of Amsterdam, Amsterdam, The Netherlands; 5 Amsterdam Cardiovascular Sciences, Amsterdam UMC, University of Amsterdam, Amsterdam, The Netherlands

**Keywords:** CKD-EPI equation, chronic kidney disease (CKD), cardiovascular prediction, SCORE2/SCORE2 Add-on, race-free kidney function estimation, multiethnic population

## Abstract

**Aim:**

To assess the impact of the race-free 2021 CKD-EPI equation compared with the 2009 CKD-EPI equation (with/without race correction) on chronic kidney disease (CKD) detection and cardiovascular (CV) risk categorization in a multiethnic European population.

**Methods:**

This cross-sectional analysis included 21,617 participants of the Healthy Life in an Urban Setting (HELIUS) study in Amsterdam: Dutch, South-Asian Surinamese, African Surinamese, Ghanaian, Turkish, and Moroccan adults aged 18–70 years. Estimated glomerular filtration rate (eGFR) was calculated using the 2009 CKD-EPI equation (with/without race coefficient) and the race-free 2021 CKD-EPI equation. CKD was defined as eGFR <60 mL/min/1.73 m^2^ and/or urinary albumin-to-creatinine ratio ≥3 mg/mmol. CV risk was assessed using SCORE2 and SCORE2 CKD Add-on models.

**Results:**

Compared with the 2009 equation, the 2021 equation yielded lower eGFR values in African Surinamese and Ghanaian participants and marginally higher values in other groups. This led to a modest increase in CKD prevalence among those of African ancestry but did not affect overall CKD classification (Cohen’s κ = 0.94–0.99). Sensitivity, specificity, and C-statistics for CKD detection in risk groups were similar across equations. Integration of CKD modestly increased CV risk estimates, but results were virtually identical across the 2009 and 2021 equations.

**Conclusion:**

In this multiethnic European cohort, the race-free 2021 CKD-EPI equation produced different kidney function estimates, but both CKD and CV risk classifications were nearly identical to the 2009 equation. These findings support their clinical interchangeability and endorse adoption of the race-free equation as an equitable and reliable standard for kidney function assessment and CV risk prediction.

**Lay Summary:**

In this large, multiethnic European cohort, adoption of the race-free 2021 CKD-EPI equation did not meaningfully alter chronic kidney disease (CKD) detection or cardiovascular (CV) risk stratification compared with the 2009 equation.

- The 2021 CKD-EPI equation lowered estimated glomerular filtration rate (eGFR) in individuals of African descent living in Europe but did not change CKD detection.
- Agreement between the 2009 and 2021 CKD-EPI equations for CKD classification was almost perfect, and CV risk categorization using SCORE2 and SCORE2 CKD Add-on tools was largely unaffected, indicating that adopting the race-free CKD-EPI equation is unlikely to alter CV risk management at the population level.

## Graphical Abstract

**Figure:**
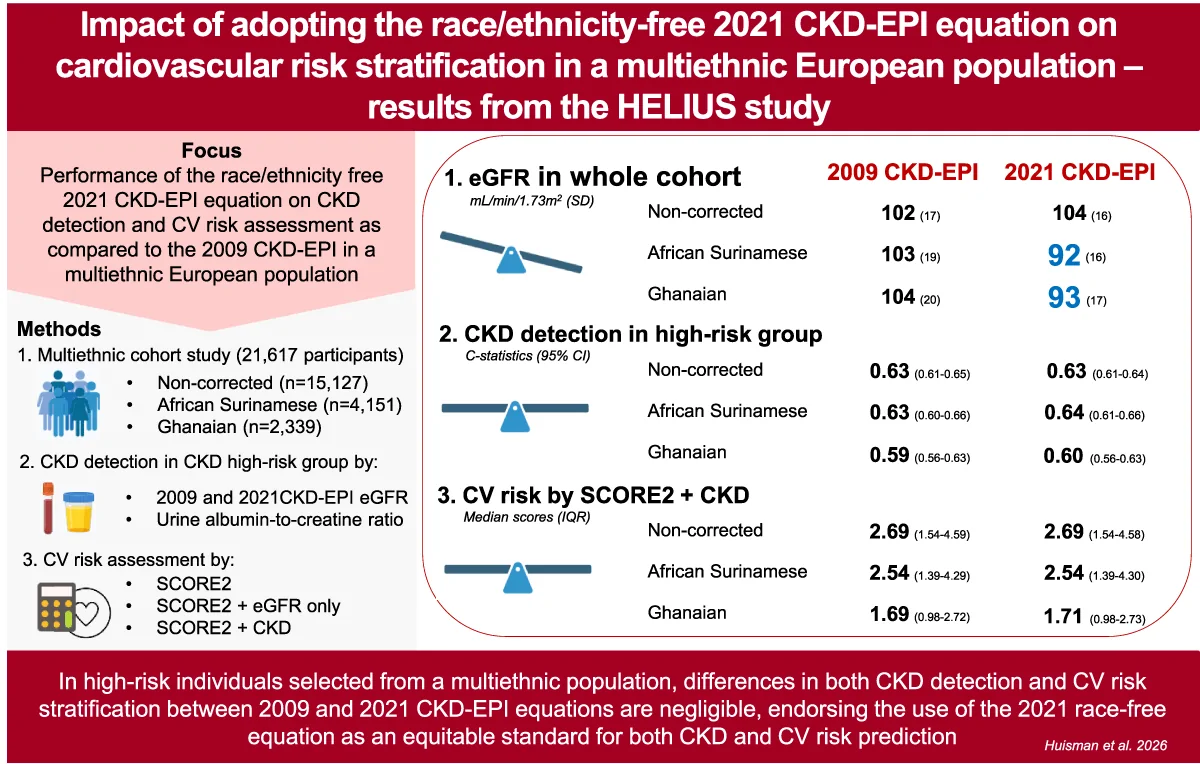


## Introduction

Chronic kidney disease (CKD) is a major determinant of cardiovascular disease (CVD) morbidity and mortality (1). Recently, guidelines (e.g., 2021 European Society of Cardiology (ESC) Guidelines) recognized CKD as an independent and causal cardiovascular (CV) risk factor, underscoring its significant role in CV risk stratification (2). However, while CKD is widely acknowledged as a key CV risk factor, the implications of the race-free 2021 CKD-EPI equation, recommended by the 2024 KDIGO guidelines, to diagnose CKD and to assess CV risk in multiethnic European populations remain largely unexplored.

In clinical practice, the diagnosis and staging of CKD is based on estimated glomerular filtration rate (eGFR) and urinary albumin excretion (3,4). The 2009 CKD-EPI creatinine equation, long recommended by KDIGO, includes a race coefficient for individuals self-identified as “Black”, that is, those of African or African-American descent, to account for higher average serum creatinine levels (5). This race variable was derived from US cohorts and, although applied across the world, may yet not be applicable for populations outside the US (6).

Importantly, the inclusion of race as a biological variable has been increasingly questioned, given that race represents a social rather than biological construct (7,8,9). In response, a revised, race-free 2021 CKD-EPI equation was introduced and subsequently endorsed by the 2024 KDIGO guidelines, which recommend avoiding the use of race in eGFR calculation (4) and aim to improve equity in CKD classification.

Previous studies have examined the impact of the race-free 2021 CKD-EPI equation, including its effect in White Europeans as well as in smaller-sized cohorts across the world comparing Black and non-Black groups, revealing potential changes in CKD classification and mortality risks (10,11). However, most evaluations of the race-free 2021 CKD-EPI equation have been performed in US cohorts focusing primarily on binary Black–White comparisons. Consequently, its performance in multiethnic European populations with more diverse ethnic and sociocultural backgrounds remains unclear, particularly regarding equitable CKD detection and CV risk assessment across ethnic groups.

Parallel to these developments, CV risk prediction models such as SCORE2 and SCORE2-OP have been updated to enable age-specific and more individualized risk estimation. The introduction of SCORE2 CKD Add-on tools, incorporating eGFR and albuminuria, further underscores the integration of renal biomarkers into CV risk prediction frameworks (12).

Therefore, the present study aims to evaluate the impact of the race-free 2021 CKD-EPI equation compared with the 2009 CKD-EPI equation (with and without applying the race correction) on CKD prevalence and CV risk categorization using the SCORE2 and SCORE2 CKD Add-on models in a multiethnic European population. We hypothesize that the removal of the race coefficient leads to a reclassification of CKD and CV risk, with implications for equitable and precise risk assessment in diverse populations.

## Methods

### Study design and population

The Healthy Life in an Urban Setting (HELIUS) study is a large, multiethnic cohort established in Amsterdam, the Netherlands, designed to examine ethnic inequalities in health. The study design and rationale have been described previously (13,14). Briefly, between 2011 and 2015, adults aged 18–70 years from the six largest ethnic groups in Amsterdam were randomly sampled through the municipal registry, stratified by country of birth and parental origin. Participants completed standardized questionnaires, underwent physical examinations, and provided biological samples. When needed, assistance from ethnically matched interviewers was offered. The overall response rate was 28%, varying across ethnic groups. It included large numbers of participants from each ethnic group and was designed using stratified sampling to capture the multiethnic composition of the Amsterdam population (13,14). The study was approved by the Academic Medical Center Institutional Review Board (METC 10/100#10.17.1729), and all participants provided written informed consent.

After exclusion of participants with missing data on CKD measures (n = 122), educational level (n = 195), or unknown ethnicity (n = 48), as well as those of Javanese Surinamese (n = 233) or other/unknown Surinamese origin (n = 267), the analytic population comprised 21,617 individuals. Missing data were handled using complete-case analysis, excluding participants with missing values on key variables. No imputation was performed. Participants were classified as Dutch (n = 4,564), South-Asian Surinamese (n = 3,043), African Surinamese (n = 4,151), Ghanaian (n = 2,339), Turkish (n = 3,614), or Moroccan (n = 3,906) in origin. For analyses aligned with KDIGO definitions, we categorized participants into three main ethnic groups:

- Non-African descent: Dutch, South-Asian Surinamese, Turkish, and Moroccan (n = 15,127).
- African Surinamese (n = 4,151).
- Ghanaian (n = 2,339).

To facilitate age-specific risk estimation, participants were stratified into three age categories: <50 years, 50–69 years, and ≥70 years (2).

### Ethnicity

Ethnic background was defined using the country of birth of participants and both parents, in line with the HELIUS study design (13). Participants were classified as Dutch when both they and their parents were born in the Netherlands. Participants were classified as of other origin when they were born abroad with at least one parent born abroad (first generation), or born in the Netherlands with both parents born abroad (second generation). Within participants of Surinamese origin, individuals were further classified based on self-reported ethnic background into African Surinamese, South-Asian Surinamese, Javanese Surinamese and other/unknown Surinamese origin.

### Kidney function and CKD definition

Serum creatinine (µmol/L) was measured enzymatically (Roche C702, C8000 platform). eGFR was calculated using three equations:

- the 2009 CKD-EPI creatinine equation (with race coefficient),
- the same equation without the race coefficient, and
- the race-free 2021 CKD-EPI equation.

Urinary albumin and creatinine concentrations were measured in early-morning spot urine samples using immunochemical and spectrophotometric methods, respectively, and used to calculate the albumin-to-creatinine ratio (ACR, mg/mmol). CKD was defined as eGFR <60 mL/min/1.73 m^2^ and/or ACR ≥3 mg/mmol, based on a single measurement.

### Cardiovascular and metabolic risk factors

Anthropometric, blood pressure, and biochemical measurements followed standardized protocols. Body mass index (BMI) was calculated as weight (kg)/height^2^ (m^2^). Blood pressure (BP) was measured twice after five minutes of seated rest; the mean was used. Hypertension was defined as systolic BP ≥140 mmHg, diastolic BP ≥90 mmHg, or use of antihypertensive medication. Diabetes mellitus was defined as fasting glucose ≥7 mmol/L or glucose-lowering medication. Hypercholesterolemia was defined as total cholesterol >6.22 mmol/L. Smoking was categorized as current versus non-current. Prevalent CVD was assessed using the Rose Angina Questionnaire and self-reported history of myocardial infarction, angina, peripheral artery disease, or cerebrovascular events. Educational level was classified into four categories ranging from primary to higher vocational/university education.

### Cardiovascular risk assessment

Ten-year CV risk was estimated using the SCORE2 algorithm according to the 2021 ESC Guidelines (2). SCORE2 was applied to participants aged <50 years and participants aged 50–69 years. SCORE2-OP was not used due to limited numbers of participants aged ≥70 years in the HELIUS cohort. Enhanced models were constructed using SCORE2 CKD Add-on tools: (i) eGFR-only (eGFR <60 mL/min/1.73 m^2^) model and (ii) combined eGFR + ACR model (CKD).

### Statistical analyses

Continuous variables are presented as mean ± standard deviation (SD) or median (IQR); categorical variables are presented as n (%). Normality was assessed using visual inspection of histograms and evaluation of skewness and kurtosis. Parametric analyses were performed after assessment of normality and homoscedasticity; non-parametric alternatives were applied when these assumptions were not met. Between-group differences were assessed using one-way ANOVA and χ^2^ tests. ACR was log-transformed prior to analysis. Paired t-tests were used to compare eGFR estimates derived from the different equations. Linear regression models (adjusted for age and sex) examined associations between ethnicity and eGFR. Differences in CKD prevalence across equations were evaluated with χ^2^ tests.

Disparities in CKD case detection when applying the different eGFR equations were calculated for all three equations by using the recommended screening approach for CKD defined by the KDIGO guideline (i.e., the presence of hypertension, diabetes mellitus, or CVD) (4). The proportions requiring screening as well as having CKD were calculated and compared between ethnic groups. Subsequently, sensitivity, specificity, and predictive values in the KDIGO CKD risk groups were calculated. In the graphical presentation, a sensitivity value ≥80% was included as a pragmatic threshold for acceptable screening performance (15,16). Agreement in CKD classification between the 2009 and 2021 CKD-EPI equations was assessed using Cohen’s κ, while discriminatory performance was evaluated using C-statistics. Differences in SCORE2 and SCORE2 CKD Add-on estimates among ethnic groups were analyzed using the Kruskal–Wallis test, followed by pairwise post hoc comparisons with Bonferroni correction. Differences in SCORE2 CKD Add-on 2009 and SCORE2 CKD Add-on 2021 algorithms within ethnic groups were analyzed using the Wilcoxon signed-rank test. Within ethnic groups, χ^2^ tests were used to assess differences in SCORE2 algorithm risk groups (i.e., low to moderate, high, and very high). Statistical significance was defined as P < 0.05. Analyses were conducted using IBM SPSS Statistics 28 and GraphPad Prism 9.5.1.

## Results

### Study population

The study cohort had a mean age of 44 years and comprised 55% women (Table 1). Participants of non-African descent were younger than those of African Surinamese or Ghanaian origin (P < 0.001). Hypertension was most prevalent among Ghanaians, who also had the highest BMI but the lowest smoking rates. Diabetes was most common among African Surinamese participants. Educational attainment was highest among African Surinamese and lowest among Ghanaians.

**Table 1:** Baseline characteristics by ethnic origin.


MEAN (SD)	OVERALL (n = 21,617)	NON-CORRECTED GROUP (n = 15,127)	AFRICAN SURINAMESE (n = 4,151)	GHANAIAN (n = 2,339)	P-VALUE

Age, years	44 (13)	43 (13)	48 (13)*	45 (11)*^†^	<0.001

<50 (%)	13,086 (60.5)	9,705 (64.2)	1,964 (47.3)*	1,417 (60.6)*^†^	<0.001

50–69 (%)	8,443 (39.1)	5,361 (35.4)	2,161 (52.1)*	921 (39.4)*^†^	<0.001

≥70 (%)	88 (0.4)	61 (0.4)	26 (0.6)	1.0 (0.0)^†^	0.005

Male sex (%)	9,129 (42)	6,608 (44)	1,616 (39)*	905 (39)*	<0.001

BMI, kg/m^2^	27.1 (5.0)	26.7 (5.2)	27.8 (5.5)*	28.5 (5.0)*^†^	<0.001

ACR, median [IQR], mg/mmol	0.28 [0.17–0.54]	0.29 [0.17–0.54]	0.26 [0.16–0.52]	0.25 [0.15–0.53]	0.790

Albuminuria ≥3 mg/mmol (%)	2,284 (10.6)	1,565 (10.3)	452 (10.9)	267 (11.4)	0.239

Smoking (%)	5,168 (24)	3,755 (25)	1,309 (32)*	104 (4.4)*^†^	<0.001

Diabetes (%)	2,334 (11)	1,568 (10)	494 (12)*	272 (12)	0.016

Hypertension (%)	7,046 (33)	3,913 (26)	1,905 (46) *	1,228 (53) *^†^	<0.001

Systolic BP, mmHg	127 (17)	128 (18)	125 (18)*	127 (18)^†^	<0.001

Total cholesterol, mmol/L	4.9 (1.0)	4.9 (1.0)	4.9 (1.0)	4.9 (1.0)	<0.001

HDL-cholesterol, mmol/L	1.4 (0.4)	1.4 (0.4)	1.5 (0.4)*	1.6 (0.4)*^†^	<0.001

Non-HDL, mmol/L	3.5 (1.0)	3.5 (1.0)	3.4 (1.0)*	3.3 (1.0)*	<0.001

Hypercholesterolemia (%)	2,210 (10)	1,563 (10)	402 (9.7)	245 (11)	0.040

CVD history (%)	3,394 (16)	2,401 (16)	663 (16)	330 (14)*	0.027

Angina pectoris (%)	1,326 (6.1)	978 (6.5)	230 (5.5)	118 (5.0)	0.016

Claudication (%)	193 (0.9)	115 (0.8)	42 (1.0)	36 (2.0)*	0.001

Possible infarction (%)	1,858 (8.6)	1,348 (8.9)	351 (8.5)	159 (7.0)*	0.014

Cerebrovascular (%)	1,119 (5.2)	763 (5.0)	261 (6.3)*	95 (4.1)^†^	<0.001

Level of education					

None or elementary (%)	3,818 (18)	2,927 (19)	231 (5.6) *	660 (28)*^†^	<0.001


*Note:* Values for categorical variables are given as a number (percentage); for continuous variables as mean (standard deviation). Non-corrected group includes participants of Dutch, South-Asian Surinamese, Turkish, and Moroccan origin, where application of a race coefficient is not advised. Abbreviations: ACR, urinary albumin-to-creatinine ratio; BMI, body mass index; BP, blood pressure; CVD history, history of cardiovascular disease (i.e., claudication intermittens, angina pectoris, myocardial infarction, and cerebrovascular accident); HDL, high-density lipoprotein; IQR, interquartile range. Post hoc analysis: *P < 0.001 vs. non-corrected; ^†^P < 0.001 vs. African Surinamese.

### Differences in eGFR estimates

Mean eGFR values differed significantly by equation and ethnicity (Supplemental Table S1). Using the 2009 CKD-EPI equation, age- and sex-adjusted eGFR was significantly higher in participants of African Surinamese and Ghanaian origin compared with participants of non-African descent (ranging from +3.2 in Ghanaian to +4.6 mL/min/1.73 m^2^ in African Surinamese participants). When the race coefficient was omitted, or the race-free 2021 CKD-EPI equation was applied, eGFR values were significantly lower in both African ancestry groups compared with participants of non-African descent, ranging from –8.9 in African Surinamese to –10.4 mL/min/1.73 m^2^ in Ghanaian participants. Among participants of non-African descent (i.e., non-corrected group), eGFR was slightly higher with the 2021 than the 2009 equation.

### CKD prevalence and agreement between equations

**Figure 1:**
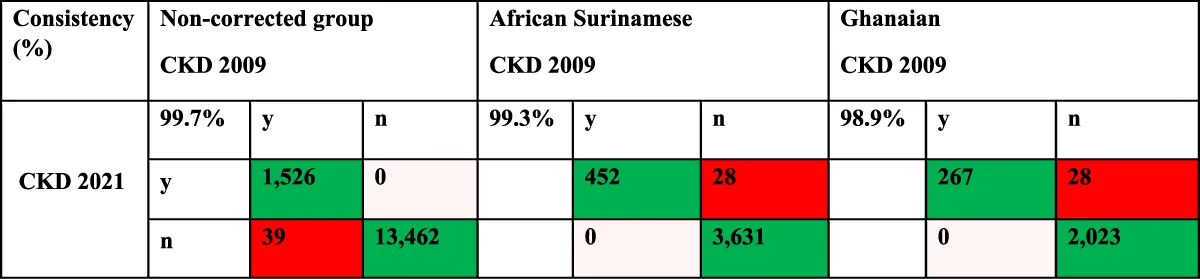
Internal consistency between CKD-EPI 2009 and 2021 in detecting CKD (y/n) per ethnic group. *Note*: Excellent internal consistency of CKD between the 2009 CKD-EPI equation and the 2021 CKD-EPI equation. Abbreviations: CKD, chronic kidney disease; n, no; y, yes.

The prevalence of CKD differed across equations. Compared with the 2009 CKD-EPI equation, use of the race-free 2021 CKD-EPI equation increased CKD prevalence among African Surinamese and Ghanaian participants, and decreased CKD prevalence among those of non-African descent (Table 2). Agreement between the 2009 and 2021 equations for CKD classification was high with Cohen’s kappa coefficients of 0.986 (0.970–1.002) in the non-corrected group, 0.966 (0.936–0.997) in African Surinamese participants, and 0.943 (0.903–0.984) in Ghanaian participants. Overall concordance ranged from 98.9% to 99.7% across groups (Figure 1).

**Table 2:** eGFR and CKD prevalence by equation by ethnic group.


	2009 CKD-EPI					2009 CKD-EPI WITHOUT RACE CORRECTION					2021 CKD-EPI				
		
	OVERALL	NON-CORRECTED	AFRICAN SURINAMESE	GHANAIAN	P-VALUE	OVERALL	NON-CORRECTED	AFRICAN SURINAMESE	GHANAIAN	P-VALUE	OVERALL	NON-CORRECTED	AFRICAN SURINAMESE	GHANAIAN	P-VALUE

eGFR estimates, mL/min/1.73 m^2^	102 (18)	102 (17)	103 (19)	104 (20)*^†^	<0.001	98 (18)^‡^	NA	89 (17)*^‡^	90 (17)*^†‡^	<0.001	101 (17)^‡^	104 (16)^‡^	92 (16)*^‡^	93 (17)*^‡^	<0.001

eGFR <60 mL/min/1.73 m^2^, n (%)	275 (1.2)	176 (1.2)	56 (1.3)	32 (1.4)	0.249	429 (1.9)^‡^	NA	147 (3.5)*^‡^	95 (4.1)*^‡^	<0.001	297 (1.3)^‡^	125 (0.8)^‡^	97 (2.3)*^‡^	67 (2.9)*^‡^	<0.001

CKD, n (%)	2,335 (10.5)	1,565 (10.3)	452 (10.9)	267 (11.4)	0.239	2,460 (11.1)^‡^	NA	523 (12.6)*^‡^	321 (13.7)*^‡^	<0.001	2,351 (10.6)^‡^	1,526 (10.1)^‡^	480 (11.6)^‡^*	295 (12.6)^‡^*	<0.001


*Note*: Values for categorical variables are given as number (percentage); for continuous variables as mean (SD/standard deviation). Abbreviations: CKD, chronic kidney disease; eGFR, estimated glomerular filtration rate. Post hoc analysis: *P < 0.001 vs. non-corrected group; ^†^P < 0.001 vs. African Surinamese; ^‡^P < 0.001 vs. 2009 CKD-EPI.

### CKD detection and diagnostic performance

**Figure 2:**
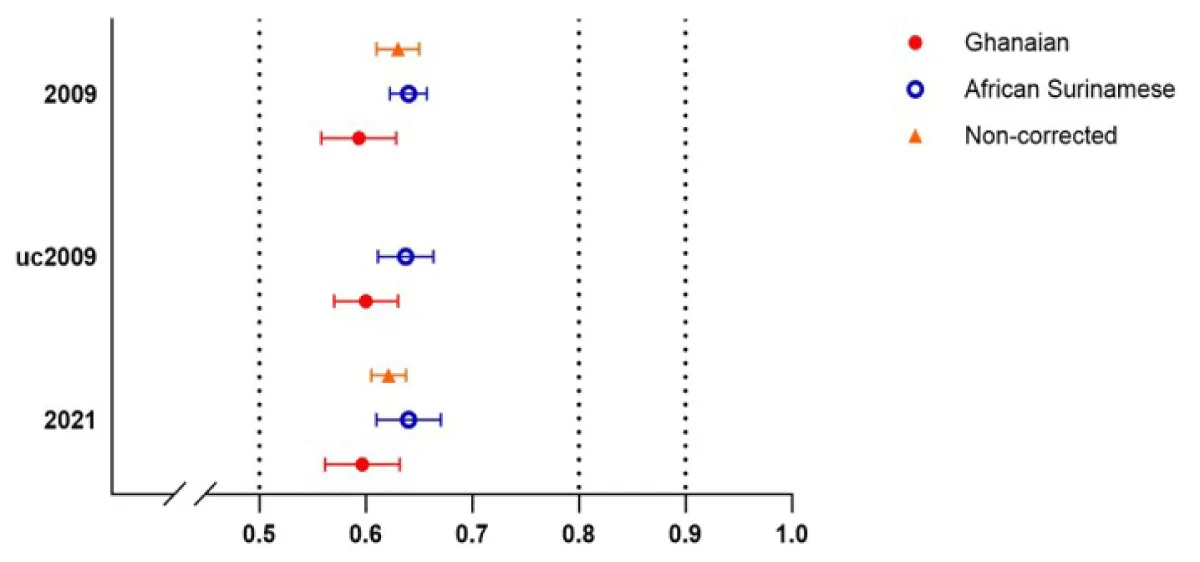
C-statistic per ethnic group per equation for detecting CKD with the traditional screening approach.* *Note*: C-statistics with 95% confidence interval (CI). Dotted line at 0.5: low diagnostic accuracy. Dotted line at 0.8: moderate diagnostic accuracy. Dotted line at 0.9: excellent diagnostic accuracy. No significant differences were found. Abbreviations: CKD, chronic kidney disease; uc, uncorrected. *Screening participants with hypertension, diabetes mellitus, and/or cardiovascular (CV) history.

Within the predefined CKD risk groups according to KDIGO, sensitivity and specificity for CKD detection were low across all ethnic groups and similar between equations (Supplemental Table S2 and Supplemental Figure S1). C-statistics for CKD were comparable across formulas (Figure 2).

### Cardiovascular risk estimation

SCORE2 risk increased with age and differed significantly by ethnicity (P < 0.001), being highest in African Surinamese and lowest in Ghanaian participants (Table 3). Incorporation of eGFR <60 mL/min/1.73 m^2^ (SCORE2 Add-on eGFR 2009 and 2021) or SCORE2 Add-on CKD (2009 and 2021, including both eGFR and ACR criteria) increased SCORE2 risk estimates, particularly among participants aged 50–69 years. Median SCORE2 risk estimates were similar between the 2009 and 2021 CKD-EPI equations across ethnic groups (Table 3). In all ethnic groups, except for the overall population, SCORE2 Add-on models using the 2021 CKD-EPI equation yielded higher risk estimates and different risk category classifications compared with models using the 2009 CKD-EPI equation (Table 4).

**Table 3:** Risk scores according to SCORE2, SCORE2 Add-on eGFR <60 mL/min/1.73 m^2^, SCORE2 Add-on CKD.


	OVERALL (n = 18,833)	NON-CORRECTEDGROUP (n = 12,691)	AFRICAN SURINAMESE (n = 3,425)	GHANAIAN (n = 2,265)	P-VALUE

SCORE2	0.93 (0.29–2.34)	0.90 (0.27–2.36)	1.24 (0.42–2.89)*	0.67 (0.24–1.55)*^†^	<0.001

<50 years	0.39 (0.15–0.96)	0.41 (0.14–1.02)	0.41 (0.17–0.95)	0.31 (0.14–0.65)*^†^	<0.001

50–69 years	2.44 (1.37–4.16)	2.66 (1.51–4.46)	2.49 (1.35–4.14)*	1.66 (0.97–2.66)*^†^	<0.001

SCORE2 + eGFR <60 mL/min/1.73 m^2^ 2009	0.93 (0.29–2.35)	0.90 (0.27–2.37)	1.24 (0.42–2.90)*	0.67 (0.24–1.57)*^†^	<0.001

<50 years	0.39 (0.15–0.96)	0.41 (0.14–1.02)	0.41 (0.17–0.96)	0.31 (0.14–0.65)*^†^	<0.001

50–69 years	2.45 (1.38–4.19)	2.66 (1.51–4.51)	2.50 (1.35–4.19)*	1.67 (0.97–2.67)*^†^	<0.001

SCORE2 + eGFR<60 mL/min/1.73 m^2^ 2021	0.93 (0.29–2.35)	0.91 (0.27–2.37)^‡^	1.24 (0.42–2.91)*^‡^	0.67 (0.24–1.58)*^†‡^	<0.001

<50 years	0.39 (0.15–0.96)	0.41 (0.14–1.02)	0.41 (0.17–0.96)	0.31 (0.14–0.65)*^†^	<0.001

50–69 years	2.46 (1.38–4.19)	2.66 (1.51–4.50)	2.50 (1.35–4.21)*	1.68 (0.98–2.70)*^†^	<0.001

SCORE2 + CKD 2009	0.95 (0.30–2.39)	0.92 (0.28–2.40)	1.26 (0.43–2.97)*	0.68 (0.25–1.60)*^†^	<0.001

<50 years	0.40 (0.15–0.98)	0.42 (0.15–1.04)	0.41 (0.17–0.97)	0.32 (0.14–0.65)*^†^	<0.001

50–69 years	2.50 (1.41–4.27)	2.69 (1.54–4.59)	2.54 (1.39–4.29)*	1.69 (0.98–2.72)*^†^	<0.001

SCORE2 + CKD 2021	0.95 (0.30–2.39)	0.92 (0.28–2.40)^‡^	1.26 (0.43–2.98)*^‡^	0.68 (0.25–1.61)*^†‡^	<0.001

<50 years	0.40 (0.15–0.98)	0.42 (0.15–1.04)	0.41 (0.17–0.97)	0.32 (0.14–0.65)*^†^	<0.001

50–69 years	2.50 (1.41–4.27)	2.69 (1.54–4.58)	2.54 (1.39–4.30)*	1.71 (0.98–2.73)*^†^	<0.001


*Note:* Median scores (IQR) for SCORE2, SCORE2 with add-on eGFR <60 mL/min/1.73 m^2^, and SCORE2 with add-on CKD, stratified by ethnicity and age group. Abbreviations: CKD, chronic kidney disease; eGFR, estimated glomerular filtration rate; IQR, interquartile range. Post hoc analysis: *P < 0.001 vs, non-corrected; ^†^P < 0.001 vs. African Surinamese; ^‡^P < 0.001 vs. 2009 CKD-EPI.

**Table 4:** Risk groups according to SCORE2 Add-on eGFR <60 mL/min/1.73 m^2^ and CKD by CKD-EPI 2009 and 2021.


N (%)	OVERALL (n = 18,833)	NON-CORRECTEDGROUP (n = 12,691)	AFRICAN SURINAMESE (n = 3,425)	GHANAIAN (n = 2,265)	P-VALUE

SCORE2, <50 years					

Low to moderate (<2.5%)	10,671 (79.9)	7,605 (78.4)	1,536 (78.2)	1,332 (94)*^†^	<0.001

High (2.5% to <7.5%)	688 (5.2)	552 (5.7)	95 (4.8)	23 (1.6)*^†^	<0.001

Very high (≥7.5%)	20 (0.1)	19 (0.2)	–	–	0.061

SCORE2, 50–69 years					

Low to moderate (<5%)	6,116 (70.1)	3,578 (66.7)	1,477 (68.3)	862 (93.6)*^†^	<0.001

High (5 to <10%)	1,118 (12.8)	782 (14.6)	266 (12.3)*	43 (4.7)*^†^	<0.001

Very high (≥10)	143 (1.6)	104 (1.9)	28 (1.3)	4 (0.4)	0.460

+eGFR <60 mL/min/1.73 m^2^ 2009, <50 years					

Low to moderate (<2.5%)	10,650 (79.8)	7,593 (78.2)	1,534 (78.1)	1,326 (93.6)*^†^	<0.001

High (2.5% to <7.5%)	706 (5.3)	562 (5.8)	96 (4.3)	26 (1.8)*^†^	<0.001

Very high (≥7.5%)	23 (0.2)	21 (0.2)	1.0 (0.1)	–	0.054

+eGFR <60 mL/min/1.73 m^2^ 2009, 50–69 years					

Low to moderate (<5%)	5,979 (68.5)	3,497 (65.2)	1,448 (67.0)	840 (91.2)*^†^	<0.001

High risk (5 to <10%)	1,203 (13.8)	823 (15.4)	288 (13.3)*	62 (6.7)*^†^	<0.001

Very high (≥10)	195 (2.2)	144 (2.7)	35 (1.6)	7 (0.8)	0.143

+ eGFR <60 mL/min/1.73 m^2^ 2021, <50 years					

Low to moderate (<2.5%)	10,641 (79.7)^‡^	7,596 (78.3)^‡^	1,528 (77.8)^‡^	1,320 (93.2)*^†‡^	<0.001

High (2.5% to <7.5%)	715 (5.4)^‡^	559 (5.8)^‡^	102 (5.2)^‡^	35 (2.5)*^†‡^	<0.001

Very high (≥7.5%)	23 (0.2)^‡^	21 (0.2)^‡^	1.0 (0.1)	–	0.061

+ eGFR <60 mL/min/1.73 m^2^ 2021, 50–69 years					

Low to moderate (<5%)	5,960 (68.3)^‡^	3,523 (65.7)^‡^	1,429 (66.1)^‡^	812 (88.2)*^†‡^	<0.001

High risk (5 to <10%)	1,227 (14.1)^‡^	809 (15.1)^‡^	301 (13.9)*^‡^	89 (9.7)*^†‡^	<0.001

Very high (≥10)	190 (2.2)^‡^	132 (2.4)^‡^	41 (1.9)^‡^	8 (0.9)^‡^	0.207

+ CKD 2009, <50 years					

Low to moderate (<2.5%)	9,653 (72.3)	6,907 (71.2)	1,391 (70.8)	1,172 (82.7)*^†^	<0.001

High (2.5% to <7.5%)	1,638 (12.3)	1,197 (12.3)	228 (11.6)	182 (12.8)*^†^	<0.001

Very high (≥7.5%)	88 (0.7)	72 (0.7)	12 (0.6)	1.0 (0.1)	0.054

+ CKD 2009, 50–69 years					

Low to moderate (<2.5%)	5,458 (62.5)	3,192 (59.5)	1,309 (60.6)	777 (84.4)*^†^	<0.001

High (2.5% to <7.5%)	1,591 (18.2)	1,036 (19.3)	394 (18.2)*	119 (12.9)*^†^	<0.001

Very high (≥10)	328 (3.8)	236 (4.4)	68 (3.1)	13 (1.4)	0.402

+ CKD 2021, <50 years					

Low to moderate (<2.5%)	9,646 (72.3)^‡^	6,909 (71.2)^‡^	1,388 (70.7)^‡^	1,166 (82.3)*^†‡^	<0.001

High (2.5% to <7.5%)	1,645 (12.3)^‡^	1,195 (12.3)^‡^	231 (11.8)^‡^	188 (13.3)*^†‡^	<0.001

Very high (≥7.5%)	88 (0.7)^‡^	72 (0.7)^‡^	12 (0.6)^‡^	1.0 (0.1)^‡^	0.054

+ CKD 2021, 50–69 years					

Low to moderate (<2.5%)	5,446 (62.4)^‡^	3,213 (59.9)^‡^	1,297 (60.0)^‡^	755 (82.0)*^†‡^	<0.001

High (2.5% to <7.5%)	1,607 (18.4)^‡^	1,023 (19.1)^‡^	402 (18.6)*^‡^	141 (15.3)*^†‡^	<0.001

Very high (≥10)	324 (3.7)^‡^	228 (4.3)^‡^	72 (3.3)^‡^	13 (1.4)^‡^	0.426


*Note:* Distribution of risk categories for SCORE2 algorithms by age group and ethnicity. Abbreviations: CKD, chronic kidney disease; eGFR, estimated glomerular filtration rate. Post hoc analysis: *P < 0.001 vs. non-corrected; ^†^P < 0.001 vs. African Surinamese; ^‡^P < 0.001 vs. 2009 CKD-EPI.

## Discussion

In this large, multiethnic European cohort, use of the race-free 2021 CKD-EPI equation altered eGFR values but had minimal impact on the detection of CKD or CV risk stratification compared with the 2009 CKD-EPI equation. Removal of the race coefficient, an important ethical and conceptual shift, resulted in higher CKD prevalence among participants of African descent, without enhancing diagnostic accuracy or predictive performance in the overall study population.

These findings are consistent with US-based studies that informed the development of the 2021 CKD-EPI equation, where analyses primarily compared two broad racial categories, Black and White participants. Our study extends these observations to a large multiethnic European cohort, including multiple ethnic groups with distinct sociocultural and ancestral backgrounds. This heterogeneity provides important insights into the performance and clinical implications of the race-free equation in a European population setting that differs substantially from the populations in which the equation was originally developed (17). The near-perfect agreement between the 2009 and 2021 equations observed in our cohort indicates that practical reclassification is uncommon despite minor numerical differences in eGFR estimates. Thus, while the transition to a race-free approach promotes fairness and clinical equity (18,19), its overall effect on CKD detection and CV risk stratification appears limited.

The present findings should be interpreted in the context of ongoing changes in eGFR reporting, particularly the removal of the race coefficient from the CKD-EPI equation. This development reflects increasing concerns regarding equity in creatinine-based estimation and supports the transition toward race-free approaches in clinical practice, as recommended by the NKF-ASN Task Force (18) and discussed in recent critical perspectives on race and GFR estimation (19). Importantly, this transition aims to improve equity while maintaining acceptable accuracy in kidney function assessment, which is supported by the high agreement observed between the 2009 and 2021 equations in our cohort (Figure 1). However, residual differences in eGFR estimates may persist even in race-free models, indicating that removal of race does not eliminate all sources of variation in creatinine-based estimation (17,20,22). In parallel, interest in alternative markers of kidney function has increased. Cystatin C becomes more acceptable as an alternative marker of kidney function because it is less influenced by muscle mass and demographic factors (4). These considerations highlight the limitations of creatinine-based eGFR, as serum creatinine is influenced by factors such as muscle mass, diet, and tubular secretion, which are not fully captured by demographic adjustments (20,21). Together, these limitations underscore the need for more individualized or multi-marker approaches, such as combining creatinine with cystatin C, to improve precision and equity in kidney function assessment (17). However, cystatin C testing remains costly and is not routinely available in many (primary) healthcare settings, particularly in resource-limited contexts, and may therefore have limited feasibility for population-wide CKD and CV risk screening strategies (22). An alternative approach to enhance kidney function assessment could involve an age-adjusted eGFR threshold, in which values are interpreted in relation to age to account for the physiological decline in kidney function over time. However, the clinical impact of this approach requires further investigation to assess its effectiveness and feasibility (23).

In our cohort, incorporating renal parameters into CV risk prediction models (SCORE2 eGFR and CKD Add-ons) did not improve overall discrimination but resulted in slightly higher risk estimates, particularly when both eGFR and albuminuria were included. This indicates that the inclusion of renal biomarkers primarily leads to upward reclassification of individuals rather than improving model performance. Importantly, the choice between the 2009 and 2021 CKD-EPI equations did not materially influence these patterns, suggesting comparable performance of both equations in population-based CV risk estimation. Taken together, these findings indicate that the two equations are largely interchangeable for population-based CV risk and kidney function assessment in multiethnic European populations.

This study has several strengths, including its large size, its multiethnic composition with substantial representation across ethnic groups (over >20,000 participants), standardized data collection, and use of contemporary ESC and KDIGO frameworks. By moving beyond the dichotomous US race paradigm, it provides a nuanced evaluation of eGFR estimation in a multiethnic European setting.

Limitations include reliance on single measurements of serum creatinine and albuminuria, which may have introduced misclassification due to biological variability and random error. The cross-sectional design limits causal inference and precludes assessment of longitudinal CV outcomes, restricting interpretation to associations. Although the overall response rate was 28% and may have introduced selection bias, stratified recruitment ensured inclusion of large numbers of participants from each ethnic group, resulting in a cohort that captures a broad representation of the ethnic diversity of the Amsterdam population (13,14). It is therefore unlikely that the response rate has materially affected the observed differences between the 2009 and 2021 CKD-EPI equations. Ethnic background was classified using the country of birth of participants and their parents, which may not fully capture sociocultural diversity within groups, leading to potential residual misclassification. Finally, as the study was conducted in a relatively young and generally healthy population, the generalizability of the findings to older individuals, high-risk populations, or transplant recipients may be limited and should be further explored.

In conclusion, adoption of the race-free 2021 CKD-EPI equation did not meaningfully alter CKD detection or CV risk stratification compared with the 2009 equation. Given the high degree of concordance between both formulas, they may be used interchangeably in clinical and epidemiological contexts. Importantly, the minimal clinical differences observed between the equations support transition to the race-free 2021 CKD-EPI equation. Removal of race from eGFR estimation may promote more equitable kidney function assessment without materially affecting CKD detection or CV risk stratification.

## Additional File

The additional file for this article can be found as follows:

10.5334/gh.1579.s1 Supplemental Data. Supplemental Tables S1 to S2 and Figure S1.

## Data Availability

Data may be obtained from a third party and are not publicly available. The HELIUS data are owned by the Academic Medical Center (AMC) in Amsterdam, the Netherlands. Any researcher can request the data by submitting a proposal to the HELIUS Executive Board as outlined at https://www.heliusstudy.nl/en/researchers/collaboration. Requests for further information and proposals can be submitted to heliuscoordinator@amsterdamumc.nl. The HELIUS Executive Board will check proposals for compatibility with the general objectives, ethical approvals, and informed consent forms of the HELIUS study, and potential overlap with ongoing work affiliated with HELIUS. There are no other restrictions to obtaining the data, and all data requests will be processed in the same manner.
