# Supplementary material for: Impact of Adopting the Race/Ethnicity-Free 2021 CKD-EPI Equation on Cardiovascular Risk Stratification in a Multiethnic European Population: Results from the HELIUS Study
Source: Glob Heart. 2026 Aug 21;21(1):62. doi: 10.5334/gh.1579 (PMC13496232; doi:10.5334/gh.1579)
Supplement: Supplementary file 1 [file gh-21-1-1579-s1.pdf]

SUPPLEMENTAL DATA

Table S1: Ethnic differences in age- and sex-adjusted eGFR by equation.

eGFR (mL/min/1.73 m^2)	2009 CKD-EPI		Uncorrected 2009 CKD-EPI		2021 CKD-EPI	
	Difference	P-value	Difference	P-value	Difference	P-value
**Non-corrected (reference)**						
African Surinamese	+4.6 ± 0.2	<0.001	–9.2 ± 0.2	<0.001	–8.9 ± 0.2	<0.001
Ghanaian	+3.2 ± 0.3	<0.001	–10.7 ± 0.3	<0.001	–10.4 ± 0.3	<0.001

Note: Differences in mean eGFR with standard error.

Abbreviations: CKD, chronic kidney disease; eGFR, estimated glomerular filtration rate.

Table S2: CKD case detection in participants with traditional cardiovascular and renal risk factors.*

	2009 CKD-EPI 2009			Uncorrected 2009 CKD-EPI			2021 CKD-EPI		
	N-c	AS	Gh	N-c	AS	Gh	N-c	AS	Gh
Sensitivity	56.8 (54.4–59.3)	72.1 (67.7–76.2)	70.8 (64.9–76.2)	NA	72.5 (68.4–76.3)	72.0 (66.7–76.8)	56.7 (54.2–59.3)	72.9 (68.7–76.8)	71.1 (65.7–76.3)
Specificity	63.7 (62.9–64.4)	47.2 (45.6–48.9)	41.7 (39.5–43.9)	NA	47.7 (46.0–49.3)	42.2 (40.0–44.4)	63.7 (62.9–64.5)	47.5 (45.9–49.2)	41.9 (39.8–44.1)
PPV	15.4 (14.4–16.2)	14.4 (12.9–15.8)	13.6 (12.4–16.2)	NA	16.8 (15.9–17.7)	16.7 (15.6–17.8)	15.0 (13.0–15.9)	15.5 (14.0–17.0)	15.1 (13.2–17.0)
NPV	92.7 (92.3–93.3)	93.2 (92.0–94.4)	91.6 (89.3–47.2)	NA	92.2 (91.2–93.2)	90.4 (88.6–48.4)	92.9 (92.4–93.4)	93.0 (91.8–94.2)	90.1 (89.0–92.7)

Note: No significant differences were found.

Abbreviations: AS, African Surinamese; CKD, chronic kidney disease; Gh, Ghanaian; N-c, non-corrected; NA; not applicable; NPV, negative predictive value; PPV, positive predictive value.

*Hypertension, diabetes mellitus, and/or cardiovascular (CV) history.

Figure S1: CKD case detection rate by ethnic group by equation using the traditional screening approach.*

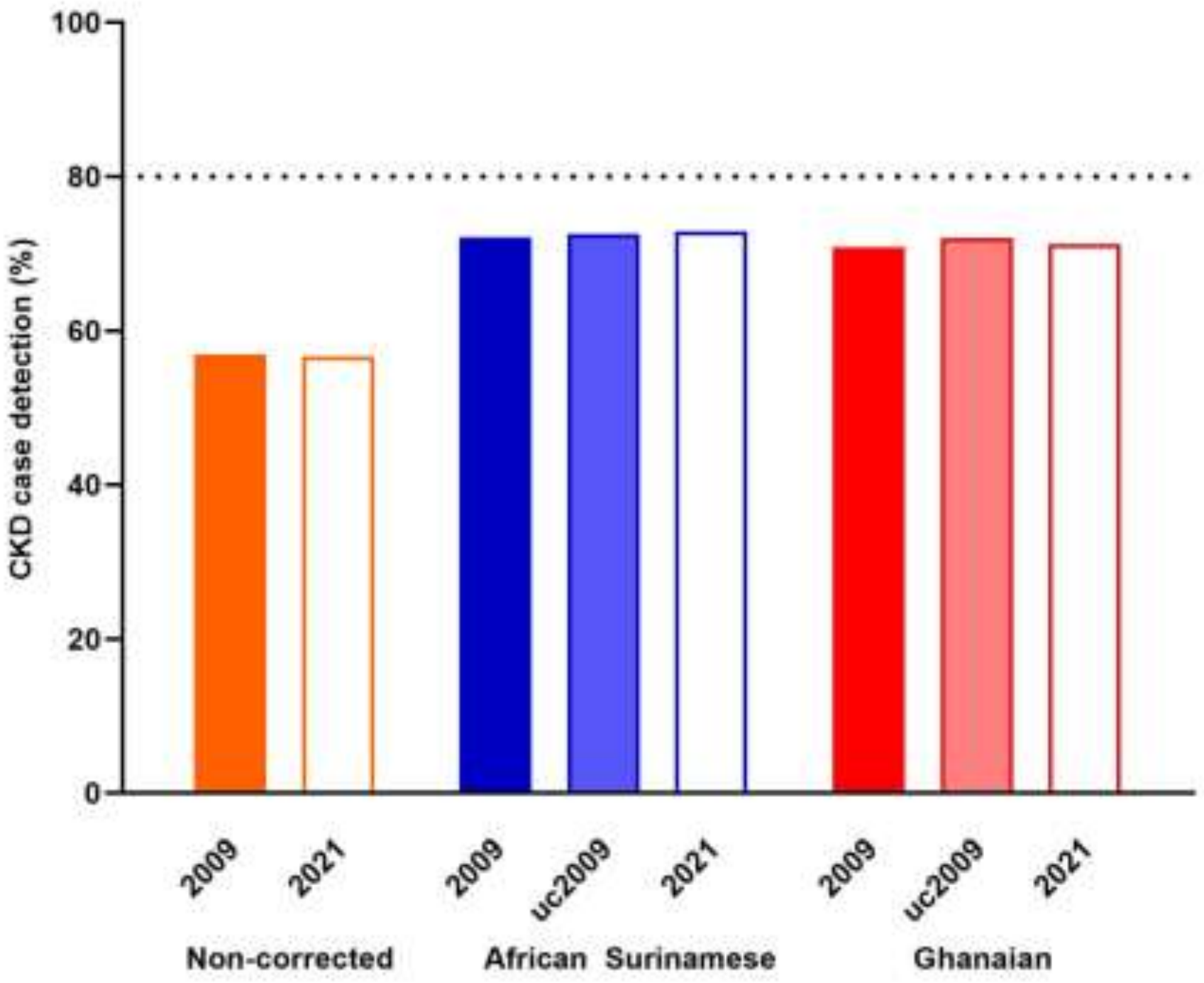

Note: Dotted line at 80: optimal screening percentage.

Abbreviations: CKD, chronic kidney disease; uc, uncorrected.

*Screening participants with hypertension, diabetes mellitus, and/or cardiovascular (CV) history.
